# Use of female-controlled dual protection methods among adolescent girls and young women living with HIV in Northern Uganda: A convergent mixed-methods study

**DOI:** 10.1371/journal.pone.0326768

**Published:** 2025-07-07

**Authors:** Edward Kumakech, Deo Benyumiza, Marvin Musinguzi, Wilfred Inzama, Doryn Ebong, James Okello, Lydia Kabiri, Jasper Watson Ogwal-Okeng

**Affiliations:** 1 Department of Nursing, Faculty of Nursing and Midwifery, Lira University, Lira, Uganda; 2 Department of Midwifery, Faculty of Nursing and Midwifery, Lira University, Lira, Uganda; 3 Department of Community Health, Faculty of Public Health, Lira University, Lira, Uganda; 4 Department of Obstetrics and Gynaecology, Faculty of Medicine, Lira University, Lira, Uganda; 5 Department of Obstetrics and Gynaecology, Lira Regional Referral Hospital, Lira, Uganda; 6 Department of Nursing, School of Health Sciences, College of Health Sciences, Makerere University, Kampala, Uganda; 7 Department of Pharmacology, Faculty of Medicine, Lira University, Lira, Uganda; Curtin University Bentley Campus: Curtin University, AUSTRALIA

## Abstract

**Background:**

Adolescent girls and young women living with HIV (AGYWLHIV) in Uganda face dual risks of HIV transmission to male partners and unintended pregnancies. Male condoms require male partner cooperation. Female-controlled dual protection methods (FCDPM) offer a potential solution by enabling AGYWLHIV to independently manage their sexual and reproductive health. This study assessed the prevalence of dual protection methods (DPMs), identified factors influencing their use, and explored reasons for non-use among AGYWLHIV in northern Uganda.

**Methods:**

A parallel convergent mixed-methods study was conducted among the AGYWLHIV attending antiretroviral therapy services at six public health facilities in Lira City and Lira District between November 2022 and April 2023. Participants were asked about the methods they were using to simultaneously prevent unintended pregnancy and HIV transmission. If not using any dual protection method, why not? Quantitative data on DPM prevalence and associated factors were analysed using descriptive statistics and chi-square tests, while qualitative data on non-use reasons were thematically analysed.

**Results:**

Among the 423 participants (median age 22 years), no one reported using the FCDPM, while only 1.2% used any form of DPM. Male condoms alone were used by 29.3% of the participants. The few who used the DPM combined the use of the male condoms with oral contraceptive pills, emergency contraceptive pills, IUD or implants. Factors positively influencing the DPM use included marital status, prior condom use, and knowledge of safer conception methods. Barriers included personal or partner disapproval of the modern contraceptives, lack of contraceptive knowledge or misconceptions, fear of contraceptive side effects or stigma, desire to conceive, and sole reliance on the male condoms.

**Conclusions:**

The findings highlight a critical gap in FCDPM uptake and the low prevalence of DPM use among AGYWLHIV. Strengthening health education on modern contraception, addressing misconceptions, and reducing fears about contraceptive side effects or stigma could improve DPM acceptance and uptake in this population.

## Background

The risk of sexual transmission of HIV in discordant or concordant relationships between adolescent girls and young women in sub-Saharan Africa (SSA) has remained a significant concern despite the wide availability of the treatment and prevention options. Adolescent girls and young women (AGYW) are defined as females aged 15–24 years. They are further subdivided into adolescent girls aged 15–19 years and young women aged 20–24 years. Despite being just 10% of the global population, AGYW disproportionally accounted for 25% of new HIV infections in the year 2020 [1].

Sexually active adolescent girls and young women living with HIV (AGYWLHIV) often with the hopes and dreams of establishing steady family and reproduction are among the key population who can propagate the sexual transmission of HIV by refusing to disclose their HIV status to their male partners (with nondisclosure prevalence ranging from 76–100%) or engaging in unprotected sexual intercourse (with unprotected sex prevalence ranging from 35–55%) often due to fear of stigma, discrimination or loss of the relationship [2–4].

Unintended pregnancy (either unwanted or mistimed) are also a common occurrence among young women in SSA with studies showing AGYWLHIV more likely to report unwanted pregnancy compared to their adult counterparts [5–7]. In fact, an institutional-based cross-sectional study conducted in South Africa reported unintended pregnancy rate at 50% among the AGYWLHIV aged 18–44 years compared to 33% among their counterpart who are not living with HIV [8]. A similar institutional-based cross-sectional study conducted in Botswana also reported a significantly higher (49%) unintended pregnancy rate among the AGYWLHIV compared to the 39% rate among their counterparts who were not living with HIV [9]. Other institutional-based cross-sectional studies among women living with HIV (WLHIV) attending HIV and AIDS care programs in south-western Uganda (aged 18–49 years) and Kenya (aged 15–19 years) reported much higher rates of unintended pregnancy at 45% and 73.9% respectively [10,11].

To address the double risks of HIV transmission and unintended pregnancy, the World Health Organization (WHO) recommends the use of dual protection methods [12]. Dual protection methods for AGYWLHIV on antiretroviral therapy (ART) include the use of condoms (male or female) alone or alongside other modern contraceptive methods whose effectiveness isn’t affected by some of the antiretroviral drugs (ARVs) [12,13]. The modern contraceptive method for women on ART includes the intrauterine devices (IUDs), and hormonal methods such as Depo-Provera, implants, or emergency contraceptive pills [13,14]. In practice, an expanded definition of dual protection method is defined as consistent male or female condom use alone or a suppressed HIV viral load alongside a modern contraceptive method preferably a hormonal contraceptive method such as Depo-Provera, implants, IUDs, or emergency contraceptive pills [15–17]. The later definition of dual protection method requires frequent HIV viral load monitoring to confirm whether the WLHIV is virally suppressed from the ART she is receiving which is impracticable in resource-constrained settings. Dual protection methods are recommended because they simultaneously prevent both HIV, other sexually transmitted infections (STIs) and unintended pregnancy [13].

Male condoms have been the mainstay dual protection method [13], but its use is low among young women in SSA due to several individual, interpersonal, and structural barriers [18]. Previous studies in Uganda reported the barriers to condom use to include partner’s disapproval or refusal, conflict with religious beliefs [19], perceived low condom efficacy [20], access challenges, lack of knowledge and misinformation about condoms, financial and socio-economic vulnerability, and alcohol consumption [21]. In fact, male condoms won’t completely be used in relationships where the male partner desires to bear children or refuses to cooperate. This was demonstrated in several previous studies that have shown that male partners who desire to bear children [4,22] were less likely to use the male condom.

Given the challenges of male condom use as a dual protection method for AGYWLHIV, female-controlled dual protection methods (FCDPM) which allows the women to take control of over their reproductive and HIV prevention needs are encouraged for the AGYWLHIV. The FCDPM for example is the use of the female condoms which allows women to protect themselves against both unintended pregnancies and STI transmissions including HIV [23]. Additionally, combining hormonal contraceptive methods such as injectables, oral pills, IUDs or implants which offer women long-term pregnancy prevention solutions with the taking of ART which is key in reducing the risk of HIV transmission through viral suppression to undetectable levels [15–17], allows women to achieve dual protection. When the viral load is undetectable, it becomes virtually impossible for the woman to transmit HIV to her partner [16]. This concept is known as U = U (undetectable = untransmittable), meaning that a woman on ART who achieves an undetectable viral suppression can have unprotected sex without passing the virus to her partner [24]. These methods are discreet and effective, allowing women to manage their reproductive health without needing input or approval from their partners.

The advantages of the FCDPM are numerous. These methods such as the use of the female condoms or hormonal contraceptives or IUDs alongside ART empower women by giving them the ability to independently protect themselves and their partners [25,26]. By having control over both contraception and HIV transmission prevention, women can make informed decisions regarding their sexual health without needing to rely on their partner’s cooperation. This sense of autonomy and control is especially important in settings where gender power imbalances or societal stigmas may otherwise limit a woman’s ability to negotiate safe sexual practices.

When the WLHIV is on both a modern contraceptive method and ART and therefore protected from both unintended pregnancy and HIV transmission, the HIV-negative male partners can even use pre-exposure prophylaxis (PrEP), a 28-day course of oral antiretroviral medication that significantly reduces their risk of HIV transmission from condomless sexual intercourse [27]. If there is any suspected HIV exposure of the HIV-negative male partner, post-exposure prophylaxis (PEP), a daily antiretroviral oral medication can be used by the HIV-negative male partner as an emergency measure to prevent the HIV transmission [28]. The use of PrEP or PEP by an HIV-negative male partner of a WLHIV who is already protected against unintended pregnancy and HIV transmission through her use of modern contraceptives and ART shifts the additional responsibility for HIV prevention to the HIV-negative partner.

Previous studies have focused on the prevalence and factors associated with dual contraceptive method use among the AGYWLHIV [29–33]. Also not examined by the previous studies were the role of pill burden, knowledge and perceptions about dual protection and safer conception methods on the use of dual protection methods among key population groups like AGYWLHIV. Also important to note is that previous research about dual protection methods had included small sample sizes of the adolescent girls and young women aged 15–24 years limiting the generalizability of their findings. Therefore, unanswered questions remained. These are:

1. How prevalent is the use of the FCDPM and other forms of dual protection methods (DPMs) among the AGYWLHIV on ART?
2. What socio-demographic, sexual and reproductive health characteristics are associated with the use of DPMs among the AGYWLHIV on ART?
3. What are the reasons for the non-use of the DPMs among the AGYWLHIV on ART?

We therefore set out to investigate the prevalence of use of the FCDPM and other forms of DPMs and the associated factors among the AGYWLHIV. We also explored the reasons for the non-use of the DPMs. The research findings will inform clinical guidelines for the integrated health education and delivery of the DPMs and ART services in a single visit approach for the AGYWLHIV attending ART clinics. The findings may also inform the development of educational materials for HIV screening, education, counselling, and treatment for people in sexual partnership with the AGYWLHIV.

### Theoretical framework

This study made use of the socio-ecological model (SEM) which posits that health behaviour such as the use of the dual protection method among AGYWLHIV may be influenced by a range of factors at multiple levels, including intrapersonal, interpersonal, health institutional, and community/societal factors [34].

At the **intrapersonal level**, several factors are thought to influence the use of the DPM. Age is considered important, as older AGYWLHIV may have been exposed to more information and experiences, which could increase their likelihood of adopting DPM as found in previous studies [29,35]. Education is another critical factor, as those with higher levels of education are more likely to understand and apply dual protection methods effectively which is consistent with previous study [36]. Also consistent with a previous study, the place of residence matters, as those living in semi-urban areas, where more health educational resources are available, may be better positioned to practice DPM [30]. Additionally, recent pregnancy experiences can affect DPM use, as AGYWLHIV who have recently been pregnant may adopt DPM to delay or space out subsequent pregnancies which is consistent with a previous study [29]. Lastly, perceptions about the benefits of DPM can strongly influence the adoption of the DPM. The AGYWLHIV who correctly perceive the benefits of DPM are likely to value its use and thus adopt it more consistently.

At the **interpersonal level**, factors such as marital status can significantly influence DPM use. Consistent with a previous study, married AGYWLHIV may have opportunities to discuss DPM with their partners, which can facilitate its adoption [30]. Also consistent with a previous study, awareness of their HIV status within a couple, whether they are discordant (one partner is living with HIV and the other is not) or concordant (both partners are living with HIV), can also encourage the use of FCDPM to minimize transmission risks [29,30,35,37,38]. Reproductive goals play an important role as well whereby AGYWLHIV who do not desire children, or whose partners do not wish to have children, may be more inclined to use DPM to avoid unintended pregnancies which is also consistent with previous studies [30,36].

**Health institutional factors** also play a critical role in the adoption of DPM. Access to family planning information, education, or counselling is another significant factor whereby AGYWLHIV who have received such information are more likely to adopt DPM which is consistent with a previous study [30]. Additionally, awareness of and access to family planning services during visits to ART clinics can encourage the use of DPM [30].

At the **community or societal level**, the legal and policy environment such as age restrictions and requirements for parental consent may act as barriers to contraceptive access for AGYWLHIV [39]. Myths and misconceptions about contraception such as the fears that contraceptives may cause infertility or harmful side effects, often discourage their use [39]. Cultural beliefs whereby societal norms discourage premarital sexual activity, creating an environment where the use of contraception by unmarried AGYW is socially frowned upon [39]. Religious beliefs whereby certain religious groups advocate for abstinence-only approaches and oppose the use of modern contraceptive methods altogether [39].

The SEM model has been used before in previous studies on the utilization of sexual and reproductive health services including modern contraceptive methods among adolescents and young people in SSA and other developing countries [39–41].

### Methods

The study design, the methods and procedures for participant recruitment, sampling, data collection and analysis were previously described by Kumakech et al., 2023 [42]. Nevertheless, they are summarized below:

### Study design

This was a parallel convergent mixed-methods design [43]. The design allowed for the determination of the prevalence, associated factors, and the reasons for non-use of the DPMs among the participants in parallel substudies.

### Study area and setting

The study was conducted at ART clinics of six public health facilities located in Lira district and Lira city northern Uganda. Lira lies at about 342 kilometres from Kampala, the capital city of Uganda.

### Study population

The study population consisted of sexually exposed AGYWLHIV aged 15–24 years who were receiving ART services. Participants were eligible for inclusion in the study if they resided in Lira City or Lira District and had been sexually active within the past year. Individuals were excluded if they were severely ill and therefore unable to participate in the study procedures.

### Sample size determination method

The study sample size was 423 AGYWLHIV. This was calculated using the Kish Leslie (1965) formula [44]. The formula assumed the Z score corresponding with 95% confidence interval of 1.96, female-controlled dual protection method prevalence p of 0.5 and precision d of 0.05 to produce a sample size of 384.16 participants. The final sample size was adjusted to 423 AGYWLHIV, accounting for a 10% non-response rate. The sample was proportionately divided across the three strata (regional referral hospital patients, health centre level IV patients and the health centre level III patients) and hence across the six facilities in accordance with the stratified random sampling principle, based on the size of the patient population in each stratum.

### Participant recruitment and sampling

Participants were recruited from the six public health facility-based ART clinics where they received their ARV drug refills. A sample of 423 AGYWLHIV was selected using stratified random sampling. The sampling frame comprising of potential participant’s names, ages, addresses, telephone numbers and the next of kins were established from the ART clinic registers of the six public health facilities. All the ART clinics have registers where they maintain the demographic characteristics and the treatment information of their HIV/AIDS patients. The list of the potential participants established from the registers was then stratified into three strata, and the samples were drawn from each of the three strata. The three strata were based on the level of health facilities providing the ART to the participants namely the referral hospital, health centre level IV (primary healthcare centre), and health centres level III (dispensaries). These strata represent study population subgroups in terms of differences in the urban or rural nature of their residential address, socio-economic status, livelihood and healthcare opportunities. The random sampling method was used to select the participants from each stratum. The stratified random sampling ensured a representative sample of participants across different levels of healthcare. The sample size was allocated to the three strata and hence across the six facilities in a proportionate to size of the patient population of the stratum whereby the stratum with the highest number of patients was allocated the highest number of participants and the vice versa.

### Participant identification

Each of the selected participants from the stratified list were identified with the assistance of the healthcare providers or the community health workers at the ART clinics during their routine visits to the clinics for ARV drug refills. Additionally, community health workers affiliated with the ART clinics reached out to some participants directly. This outreach involved phone calls and or home visits to invite them to the ART clinics specifically to participate in the study. The home visits by the community health workers ensured follow up and study participation of some of the selected participants without phone and those who were not yet due for the ART clinic revisit for drug refill at the time of study.

### Data collection tool

The data collection tool used in this study was a semi-structured questionnaire, which included both closed-ended and open-ended questions. The closed-ended questions gathered data on the use of the DPMs, exposure factors and covariates likely to be associated with their adoption, while the open-ended questions explored participants’ reasons for non-use, as well as their perceptions of dual protection and safer conception methods (SCMs). The questionnaire also included items related to the participant’s socio-demographic, sexual and reproductive health characteristics.

The questionnaire was specifically developed for this study, drawing on insights from previous research on the use of dual protection methods, including condoms and modern contraceptive methods among the AGYWLHIV. The previous studies on the use of dual contraceptives or dual protection methods among the women living with HIV that provided items for developing the questionnaire for this study included studies conducted in Ethiopia [30,32,33,45–48], Nigeria [49], South Africa [4,38], Botswana [50], Brazil [36], Thailand [51], Australia [52] and Canada [29]. The researchers adapted the items to assess the participants’ awareness of and access to the family planning services offered during ART clinic visits, as well as their perceptions of the advantages and disadvantages of the DPMs and SCMs.

To ensure the clarity and relevance of the questions, the questionnaire was pretested with a ten   sample size of AGYWLHIV attending Lira University Hospital. Feedback from this pretest was used to refine and improve the questionnaire items. The feedback obtained from the pre-test participants were not highly variable which pointed to the sufficiency of the pre-test sample size.

### Data collection

Data collection was conducted from November 20, 2022, to April 30, 2023. Six research assistants, all with midwifery backgrounds, carried out the data collection after receiving comprehensive training on the study procedures and ethical considerations. The questionnaires were administered by the interviewer in the local language Langi or English for those who could not speak Langi language. Each questionnaire took approximately one hour to complete. During the data collection period, the six research assistants worked in parallel, interviewing about 10–12 participants per day.

The questionnaire administration by interviews were conducted in private rooms within the six public health facility-based ART clinics to ensure privacy and confidentiality. The data collection from the participants took place after they were seen by the health professionals. The questionnaire was pre-coded, allowing research assistants to record responses in pen directly on the questionnaire forms, ensuring a standardized approach to data collection.

### Measures of variables

#### Outcome variables.

The primary outcome variable was the use of the FCDPM. To assess this, AGYWLHIV were asked, “what methods under their control do they use to protect against both unintended pregnancy and HIV transmission to their male sexual partners during sex?” Those who reported using FCDPM were further queried about their current usage, whether they used it during their last sexual encounter, in the past three, six or 12 months. The participant who didn’t report the use of the method at all or skipped its use for over 12 months’ period were regarded as null or inconsistent users. Additionally, participants were asked to describe how exactly they used the method to evaluate the appropriateness of its use.

Since all the participants were on the ART for viral suppression, the ones classified as FCDPM users were the ones who reported: (a) using the female condoms alone or (b) using the female condoms alongside modern contraceptive methods such as the hormonal contraceptives or IUDs. The ones who reported using the natural family planning methods such as the safe days, moon beads, lactational amenorrhea, and cervical mucus viscosity monitoring without the female condoms were considered as the FCDPM non-users. This operationalization of the FCDPM use is similar to the ones used in the previous studies by Kaida et al [53] and Lawani et al [49] which were defined as the concurrent use of a barrier contraceptive method (condoms) alongside modern contraceptive method. Participants who indicated they were not using FCDPM were asked to give their reasons for non-use which facilitated a deeper understanding of the barriers faced.

To evaluate the prevalence of unprotected sexual intercourse among these sexually active AGYWLHIV, participants were asked whether they had recently engaged in sexual intercourse without any form of protection, including condoms. They were also inquired about the current number of male sexual partners they had.

To determine the prevalence of unintended pregnancies, AGYWLHIV were asked if they had become pregnant in the past year and whether the pregnancy was intended or unintended. If it was unintended, they were further questioned to ascertain whether it was mistimed or unwanted.

Similarly, to measure the prevalence of male dual protection (male condom) use, AGYWLHIV were asked about the methods they relied or depended on their male partners to prevent both unintended pregnancy and HIV transmission during sexual intercourse. If they reported using a male-dependent dual protection method, they were prompted to describe how they used it and whether they had utilized it during their most recent sexual encounter, past one, six or twelve months. Participants who confirmed using the male condoms alongside other modern contraceptive methods such as oral contraceptive pills, emergency contraceptive pills, injectables, implants or IUDs were categorized as using the male dual protection method. However, responses indicating that the male partner was merely taking ARV medication, PrEP, or PEP were not classified as appropriate male dual protection, as these alone do not provide comprehensive protection against both HIV transmission and unintended pregnancy. The operationalization of the male dual protection (male condom use) was close to the one used in a previous study by Tsuyuki et al [36] who defined it as consistent (always) use of the condoms

The use of DPMs which typically involves concurrent use of a barrier method (such as the male or female condoms) alongside another modern contraception method (such as oral contraceptive pills, emergency contraceptive pills, implants, injectables or IUDs) was another outcome variable in this study. To measure this, the participants who reported concurrent use of either male or female condoms alongside other modern contraceptive methods were counted.

Whereas the above three previous studies by Kaida e tal, Lawani et al, and Tsuyuki e tal [29,36,49] used to operationalize the FCDPM, their definitions of the male dual protection and dual protection methods in general were not categorically clear on the use female condoms or male condoms alone as a dual protection method compared to the work by Berer et al [54] that provided a comprehensive list of approaches for achieving dual protection. These included (a) the use of male or female condoms alone, including for vaginal and anal sex and male condoms for oral sex; (b) the use of male or female condoms plus a diaphragm or cervical cap; (c) the use of male or female condoms plus a non-barrier contraceptive such as the oral contraceptive pill, implant, injectable, patch, vaginal ring or IUD (the latter in the absence of any STIs) or male or female sterilisation.; (d) the use of the male or female condoms with the back-up of emergency contraceptive pill, induced abortion, PEP or PrEP. This comprehensive list provides an expanded definition of dual protection methods.

#### Explanatory variables.

The participant’s socio-demographic characteristics such as marital status, having rural or urban residential address, religion, educational level, occupation or substance use were among the explanatory variables in this study. To assess the socio-demographic characteristics of the AGYWLHIV, participants were asked to provide information about their age, ethnicity, residential address, marital status, religion, educational level, source of income, occupation, monthly income, distance from the nearest health facility, and substance use behaviors, including alcohol and tobacco. To operationalize rural or urban address, women who reported to be residing in a trading centre, town, municipality or city were regarded to be having urban address and vice versa. The response options for these socio-demographic items are summarized in Table 1 and detailed in the supporting material (S1 File).

**Table 1:** Socio-demographic, sexual and reproductive health characteristics of the participants.

Sexual and reproductive factors.	F (%)
*Median age in years (IQR)*	22 (IQR 20–24)
*age group in years.* 15–19. 20–24.	99 (23.4) 324 (76.6)
*Religion.* Christian Catholics. Christian Other Denominations Islam	184 (43.5) 189 (44.7) 50 (11.8)
*Current marital status.* Single. Married.	220 (52.0) 203 (48.0)
*level of education.* No or Primary education. Secondary education. Tertiary education or higher.	258 (61.0) 125 (29.5) 40 (9.5)
*monthly income in Uganda shillings (USD equivalent).* ≤87,600 (≤ 24). >87,600 (> 24).	261 (61.7) 162 (38.3)
*residential address.* Rural area. Semi-urban or urban.	80 (18.9) 343 (81.1)
*alcohol use.* Never. Uses.	372 (87.9) 51 (12.1)
*additive drug use.* Never. Uses addictive energy drinks and herbs.	418 (98.8) 5 (1.2)
*pill burden.* 1-2 types of medicines. 3 or more types of medicines.	412 (97.4) 11 (2.6)
*median age at sexual debut (IQR).*	17 [16–18]
*age group at sexual debut.* ≤15 years. 16–19 years. 20–24 years.	105 (24.8) 259 (61.2) 59 (14.0)
*sexual debut period.* Within 1 year Within 2–5 years 6 or more years	29 (6.9) 229 (54.1) 165 (39.0)
*Gravida.* Nulligravida Gravida 1+	155 (36.6) 268 (63.4)
*Parity.* Nullipara Para 1+	200 (47.3) 223 (52.7)
*miscarriages and abortions.* 0 1+	367 (86.8) 56 (13.2)
*woman’s number of living children.* 0 1+	197 (46.6) 226 (53.4)
*whether the woman desires to bear children.* No. Yes.	47 (11.1) 376 (88.9)
*woman’s desired number of children.* 0 1-2 3-4 5+	47 (11.1) 110 (26.0) 239 (56.5) 27 (6.4)
*whether the male sexual partner’s desire to bear children.* No. Yes. Don’t know or not sure.	15 (3.5) 307 (72.6) 101 (23.9)
*whether the AGYWLHIV has recently gotten pregnant.* No. Yes.	323 (76.4) 100 (23.6)

USD is United States Dollars; F is frequency count: AGYWLHIV is adolescent girls and young women living with HIV; Km is kilometers; f is frequency count; % is percentage; < is less than, and > is greater than; IQR is the interquartile range.

The participant’s sexual and reproductive health characteristics were also considered to be among the explanatory variables in this study. To assess the sexual and reproductive health characteristics of the AGYWLHIV, participants were asked about their age at sexual debut, gravidity, parity, history of miscarriages and abortions, number of living children, desire to have children, desired number of children, their male partner’s desire to have children and his desired number of children, current pregnancy status, breastfeeding status, awareness of their HIV status, initial HIV status at the start of the current sexual relationship, whether the male partner disclosed his initial HIV status, the male partner’s initial HIV status, current discordant or concordant status, whether they disclosed their HIV status to their male partner, whether they experience sexual feelings, whether she has engaged in unprotected sexual intercourse as a AGYWLHIV and whether she has gotten pregnant in the past 1 year or was currently pregnant at the time of the study.

Another key explanatory variable for the use of the FCDPM were the women’s knowledge and perceptions about the modern contraceptive methods, dual protection methods and the safer conception methods for the WLHIV. These were measured by asking the women to report their knowledge and perceptions about the contraceptive methods, how they should be used, their benefits (advantages) and disadvantages. Other key explanatory variables on which the participants were asked to make a self-report using the interviewer-administered questionnaire were their duration on ART, the pill burden, whether they ever used the male dual protection method (male condom), and whether they used it at the last sex, their knowledge about the modern contraceptive methods offered to WLHIV from the ART clinic, whether they ever received modern contraceptive method information, education or counselling from the ART clinic, whether they ever received modern contraceptive methods and the specified method types from the ART clinics where they obtain their ARV drug refills in a single visit approach. The response options for these explanatory variables are summarized in Table 2 and included in the supplementary material (the questionnaire).

**Table 2:** Use of the Dual protection methods by the HIV discordant or concordant status of the couples.

Use of Dual protection methods	HIV discordant or concordant status			
**Female positive, Male positive**	**Female positive, Male negative**	**Female positive, Male unknown**	**Overall**
**F (%)**	**F (%)**	**F (%)**	**F (%)**
No contraceptive method but on daily ARVs for ART.	98 (50.3)	61 (41.5)	41 (50.6)	200 (47.3)
Emergency contraceptive pills (ECPs) alongside daily ARVs for ART.	5 (2.6)	8 (5.4)	3 (3.7)	16 (3.8)
Safe days alongside daily ARVs for ART.	1 (0.5)	0 (0.0)	0 (0.0)	1 (0.2)
Aspirin alongside ARVs for ART.	1 (0.5)	2 (1.4)	0 (0.0)	3 (0.7)
Sayana Press alongside ARVs for ART.	6 (3.1)	0 (0.0)	3 (3.7)	9 (2.1)
Daily pills (COC or POC) alongside ARVs for ART.	4 (2.1)	11 (7.5)	2 (2.5)	17 (4.0)
3-monthly Depo-Provera injection alongside ARVs for ART.	39 (20.0)	23 (15.6)	15 (18.5)	77 (18.2)
Implant alongside ARVs for ART.	21 (10.8)	9 (6.1)	7 (8.6)	37 (8.7)
IUD alongside ARVs for ART.	8 (4.1)	2 (1.4)	1 (1.2)	11 (2.6)
COC or POC pills after sex alongside ARVs for ART.	7 (3.6)	23 (15.6)	5 (6.2)	35 (8.3)
Male condoms alongside ARVs for ART.	2 (1.0)	2 (1.4)	2 (1.5)	6 (1.4)
COC or POC pills before and after sex alongside ARVs for ART.	1 (0.5)	0 (0.0)	1 (1.2)	2 (0.5)
COC or POC pills before sex alongside ARVs for ART.	0 (0.0)	4 (2.7)	0 (0.0)	4 (0.9)
Male condoms and ECPs alongside ARVs for ART.	0 (0.0)	1 (0.7)	0 (0.0)	1 (0.2)
Male condoms and COC or POC pills after sex alongside ARVs for ART.	0 (0.0)	1 (0.7)	0 (0.0)	1 (0.2)
Male condoms and implants alongside ARVs for ART.	2 (1.0)	0 (0.0)	1 (1.2)	3 (0.7)
Female condoms alongside ARVs for ART.	0 (0.0)	0 (0.0)	0 (0.0)	0 (0.0)
Female condoms alongside modern contraceptive methods such as hormonal contraceptives or IUDs	0 (0.0)	0 (0.0)	0 (0.0)	0 (0.0)
Modern contraceptive method alongside viral suppression (HIV-RNA plasma viral load suppression fewer than 50 copies per milliliter) self-reported.	0 (0.0)	0 (0.0)	0 (0.0)	0 (0.0)
Modern contraceptive method by the WLHIV alongside PEP by the HIV-negative male partner.	0 (0.0)	0 (0.0)	0 (0.0)	0 (0.0)
Modern contraceptive method by the WLHIV alongside PrEP by the HIV-negative male partner.	0 (0.0)	0 (0.0)	0 (0.0)	0 (0.0)
**Total**	**195**	**147**	**81**	**423**

AGYWLHIV is woman living with HIV; F is frequency count; f is frequency; % is percentage; ARVs is antiretroviral drugs; ART is antiretroviral therapy; ECP is emergency contraceptive pills; COC is combined oral contraceptive pills; POC is progesterone only oral contraceptive pill; Df is degree of freedom; IUD is intrauterine device; PrEP is the pre-exposure prophylaxis and PEP is the post-exposure prophylaxis.

#### Covariates.

The women’s years of sexual experience measured as age at sexual debut and or the period since sexual debut was the only covariate considered in the study. These were also later converted into categorical variables as reported in Table 1 and 3. This was because women with more years of sexual experience are likely to have engaged more frequently with healthcare providers. These interactions provide opportunities for information, educational and communication programs, health campaigns, or conversations with the health providers and learn about modern contraceptive methods and also gain access to them.

**Table 3:** Socio-demographic, sexual and reproductive health factors associated with the use of the dual protection methods.

Factors	Inappropriate practice F (%)	Appropriate practice F (%)	X^2^	DF	P value
*Age group.* 15-19 years 20-24 years	98 (23.4) 320 (76.6)	1 (20.0) 4 (80.0)	0.856	1	0.856
*Educational level.* No or primary education. Secondary education Tertiary education	257 (61.5) 121 (28.9) 40 (9.6)	1 (20.0) 4 (80.0) 0 (0.0)	5.811	2	0.055
*Net monthly income in US$.* <24 24 or more	260 (62.2) 158 (37.8)	1 (20.0) 4 (80.0)	3.724	1	0.054
*Marital status.* Single Married	220 (52.6) 198 (47.4)	0 (0.0) 5 (100.0)	5.484	1	**0.025**
*Address.* Rural Semi-urban	80 (19.1) 338 (80.9)	0 (0.0) 5 (100.0)	1.180	1	0.277
*Alcohol use.* Never Drinks	367 (87.8) 51 (12.2)	5 (100.0) 0 (0.0)	0.694	1	0.405
*Drug use.* Never Uses drugs	413 (98.8) 5 (1.2)	5 (100.0) 0 (0.0)	0.061	1	0.806
*Pill burden.* 1-2 pill types 3 or more pill types	409 (97.8) 9 (2.2)	3 (60.0) 2 (40.0)	27.942	**1**	**<0.001**
*Age at sexual debut.* ≤15 years 16-19 years 20-24 years	103 (24.6) 256 (61.2) 59 (14.1)	2 (40.0) 3 (60.0) 0 (0.0)	1.801	2	0.406
*Sexual debut period.* Within 1 year Within 2–5 years 6 or more years	28 (6.7) 229 (54.8) 161 (38.5)	1 (20.0) 0 (0.0) 4 (80.0)	7.961	2	**0.019**
*HIV status of the couple.* All positive Discordant (female positive male negative) Discordant (female positive male unknown)	192 (46.2) 145 (34.7) 80 (19.1)	2 (40.0) 2 (40.0) 1 (20.0)	0.083	2	0.959
*Whether the woman desires to bear children.* No Yes	47 (11.2) 371 (88.8)	0 (0.0) 5 (100.0)	0.632	1	0.462
*Whether the male partner desires to bear children.* No Yes	11 (3.5) 307 (96.5)	0 (0.0) 5 (100.0)	0.179	1	0.672
*Whether received family planning method information, education, or counselling from health workers.* No Yes	189 (45.2) 229 (54.8)	1 (20.0) 4 (80.0)	1.270	1	0.260
*Whether aware of family planning methods offered from the ART clinic where she obtains her ARV drug refills from?* Unaware Aware	204 (48.8) 214 (51.2)	3 (60.0) 2 (40.0)	0.248	1	0.619
*whether ever received family planning methods from the ART clinic where she obtains her ARV drug refills from?* No Yes	284 (67.9) 134 (32.1)	3 (60.0) 2 (40.0)	0.143	1	0.705
*Whether the woman knows at least one correct safer conception method for AGYWLHIV?* Don’t know. Knows	198 (47.4) 220 (52.6)	0 (0.0) 5 (100.0)	4.453	1	**0.035**
*Whether the woman used the male dual protection method (male condoms) in the past 1 year?* No Yes	298 (71.3) 120 (28.7)	1 (20.0) 4 (80.0)	6.273	1	**0.012**
*Whether the woman perceives benefits of dual protection methods?* No Yes, correct benefits perceived. Unsure	28 (6.7) 331 (79.2) 59 (14.1)	0 (0.0) 5 (100.0) 0 (0.0)	2.318	2	0.314
*Whether woman ever got pregnant in the past 1 year or is currently pregnant?* No Yes	317 (75.8) 101 (24.2)	5 (100.0) 0 (0.0)	1.587	1	0.208

F is frequency count; % is percentage; ARVs is antiretroviral drugs; ART is antiretroviral therapy; US$ is United States Dollar; X^2^ is Chi-square; Df is degree of freedom; P is significance level.

### Data management and analysis

Data entry was performed using EpiData 3.1, with a double-entry validation process implemented to minimize errors associated with data entry. After validation, the data were exported to the Statistical Package for the Social Sciences (SPSS) version 26.0 for statistical analysis. Descriptive statistics were employed to determine prevalence rates, while Chi-square tests were utilized to identify associated factors related to the outcomes. Multivariate analysis for predictors of the use of the DPM was not conducted due to extremely low prevalence in this study, with only 5 participants (1.2%) reporting the DPM use. Nevertheless, for the bivariate analysis, a significance level of p < 0.05 and a 95% confidence interval were applied to all statistical tests to assess the robustness of the findings.

The qualitative responses to the open-ended questions (qualitative data) were manually analysed using a thematic analysis approach [55]. The process began with familiarization with the data. At this stage, the researchers immersed themselves in the data by transcribing the qualitative data and carefully reading the transcripts multiple times. This allowed a deep understanding of the content and for noting of the initial impressions, recurring ideas, or patterns that emerged.

After familiarization with the data, the next step involved generating initial codes. This process entailed systematically reviewing the data to identify and label meaningful segments of text that provided relevant answers to the research questions (coding). The coding was done manually, and the process was inclusive and flexible.

After coding, the researchers progressed to searching for themes. This involved grouping the codes into broader categories that represent recurring patterns or central ideas across the dataset. Themes went beyond individual codes to reflect larger, more meaningful patterns. At this stage, the researchers began exploring relationships between codes and starts to conceptualize themes that represent the overarching narratives within the data. The themes captured both explicit ideas expressed directly by the participants or implicit meanings underlying their responses.

The identified themes were then subjected to review and refinements. During this step, the researchers ensured the themes were coherent, consistent, and relevant to the research questions. This involved revisiting the dataset to confirm that the themes accurately represented the data, and no significant details were missing. Themes that overlapped or were too broad were merged, while those lacking sufficient supporting data were redefined. The goal was to ensure that the themes were both robust and reflective of the dataset.

After the themes have been refined, the next step was defining and naming the themes. The researchers clearly articulated what each theme represented and how it contributed to answering of the research questions. Each theme was carefully defined to clarify its boundaries, including what was included and excluded. Concise and descriptive names were then assigned to each theme to convey their meaning effectively to the readers.

The qualitative data analysis process ended with presentation of the findings in a clear, coherent, and compelling narrative. This involved selecting illustrative excerpts (direct quotes) from the data to support each theme and integrating these with an analysis that links the themes back to the research questions and the theoretical framework. The report offered a thoughtful interpretation of the data.

Throughout the thematic analysis process, the researchers were reflective to acknowledge the researcher’s influence on the analysis, as their perspectives and decisions inevitably shaped the outcomes. Additionally, thematic analysis was iterative rather than strictly linear whereby the researchers revisited the earlier steps to refine their understanding or adjust. Transparency was also critical, as documenting the decisions made during the analysis enhances the rigor and reproducibility of the findings.

### Ethical considerations

This study received ethical approval from the Gulu University Research Ethics Committee (GUREC), under approval number GUREC-2022–309. Written informed consent was obtained from all participants aged 18 and above. For participants under 18 years of age, assent was obtained in conjunction with written informed consent from their parents or guardians. The consent and assent processes were conducted through either a written signature or thumbprint, ensuring clarity and understanding. Research Assistants facilitated and moderated the informed consent process to ensure adherence to ethical standards.

The study adhered to the principles outlined in the Declaration of Helsinki and followed all other relevant guidelines and regulations for research involving human participants. To acknowledge their time and contribution, each participant received compensation of 10,000 Ugandan shillings (equivalent to approximately three U.S. dollars).

## Results

### Response rate

**Fig 1:**
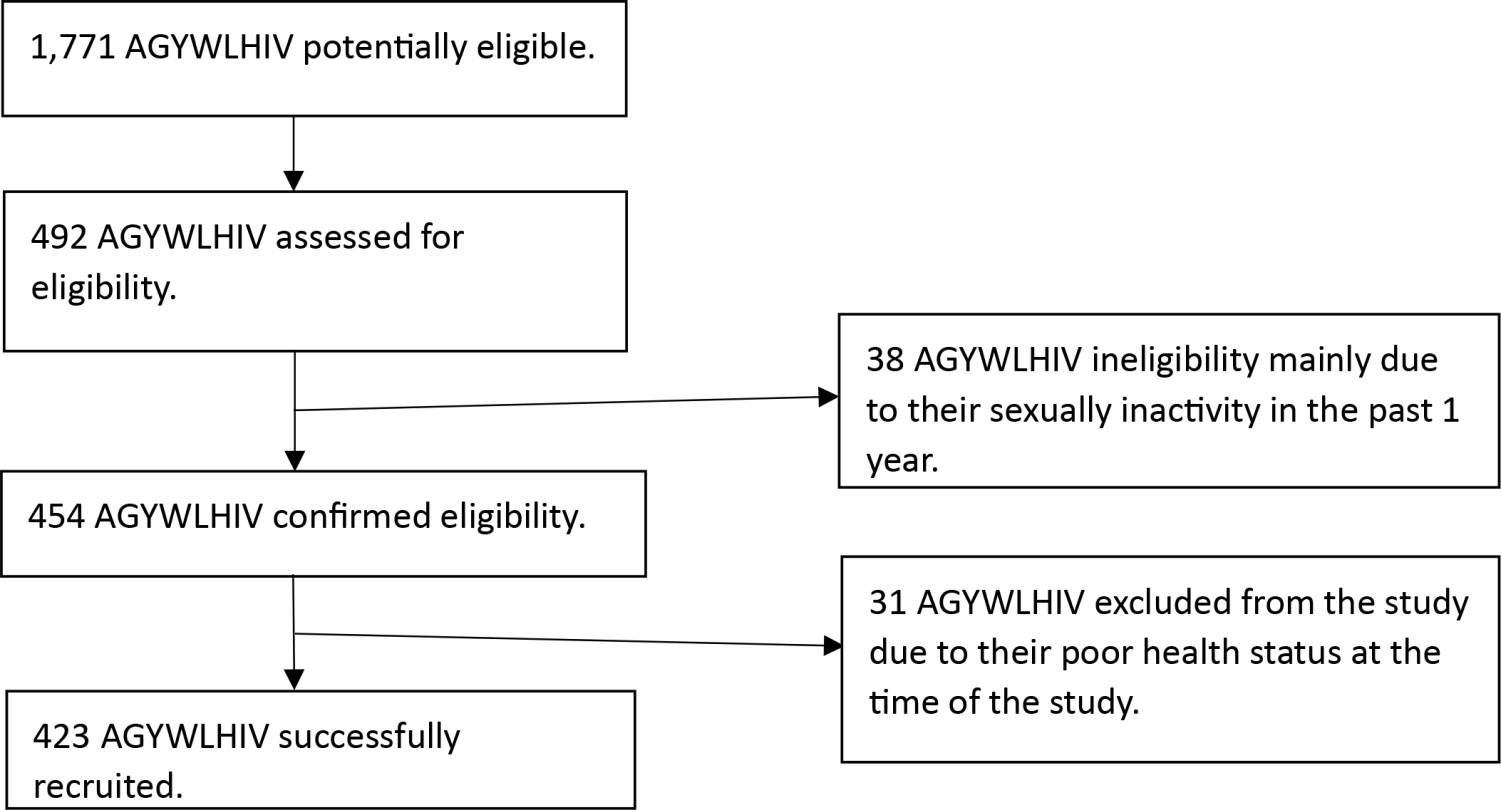
Flow chart showing participant recruitment process.

As illustrated in Fig 1, a total of 423 AGYWLHIV were successfully recruited into the study, representing a response rate of 93.2% of the targeted sample size of 454. Out of the targeted 454 participants, a total of 31 potential participants declined to participate, which accounted for 6.8%. The primary reason cited was poor health status, which prevented them from engaging in the study procedures. Notably, there were no statistically significant differences in the socio-demographic characteristics between the AGYWLHIV who participated and those who opted out. Additionally, there were no instances of missing at random data, as the questionnaire was administered by interviewers who ensured that all relevant questions were answered by each participant.

### Socio-demographic, sexual and reproductive health characteristics of the participants

Table 1 presents the descriptive analysis of the socio-demographic characteristics of the AGYWLHIV included in the study. The participants’ ages ranged from 15 to 24 years, with a median age of 22 years (interquartile range of 20–24). Most participants were aged between 19 and 24 years, with a significant proportion being married. Most had completed only primary education and were classified as low-income earners, primarily relying on domestic remittances. Additionally, most participants lived within a five-kilometre radius of a health facility.

Regarding sexual activity, all participants reported being sexually exposed, with a mean interval of recent sexual activity of 8.0 days (±15.7). Among them, 48% were married or cohabiting with their male partners. The average duration of sexual relationships with their current male partners was 2.4 years (±2.4), with participants typically initiating sexual activity with the current male partner at an average age of 19.0 years (±2.6). Notably, 88.9% of participants desired to bear children.

### Antiretroviral therapy (ART) use

The study findings indicate that at the outset of the current sexual relationship, 303 (71.6%) of the AGYWLHIV were on ART and the remaining 120 (28.4%) were not ART. Of course, all the participants [423 (100%)] were on ART at the time of the study. Just above half of the AGYWLHIV (231, 54.6%) have been on ART for more than one year. The rest 192 (45.4%) were on ART for one or less year.

### HIV discordance and positive concordance rates

The study findings indicate that from the outset of their sexual partnerships, 73 (17.3%) of the participants were concordant couples living with HIV, 51 (12.1%) were concordant couples not living with HIV, 159 (37.6) were HIV discordant couples which warranted considerations for safer conception practices and the remaining 140 (33.1%) had indeterminate status because one or both partners had unknown HIV status. Of the 159 discordant couples, 135 (84.9%) were WLHIV with negative male discordant couples and the remaining 24 (5.1%) were HIV-negative female with positive male couples.

### Unprotected sexual intercourse rate

Of the 423 AGYWLHIV in the study, most of them (332, 78.5%) engaged in a recent unprotected sexual intercourse without any protection including condoms within the past 12 months. The unprotected sexual practices were less rampant (43.1%) among the AGYWLHIV without knowledge about safer conception methods compared to 56.9% among those who knew at least one correct safer conception method (X^2^ 8,652, df 1, p 0.003). The unprotected sexual intercourse was not associated with the fertility desires of partners, awareness about providers of family planning methods, access to information about family planning methods from media sources (radio, television, posters, billboards, healthcare providers or ART clinics), or misconceptions about contraceptives including condoms.

### Changes in HIV discordance and positive concordance rates

The study findings indicate that the proportion of the concordant couples living with HIV increased to 195 (46.1%) couples, 147 (34.8%) were HIV discordant couples and 81 (19.1%) remained couples with indeterminate status because the HIV of the male partners remained unknown to the AGYWLHIV in the study.

### Unintended pregnancy rates

Out of the 423 AGYWLHIV who responded to the question on pregnancy history, 101 (23.9%) reported becoming pregnant in the past 12 months or 1 year. Among these 101 respondents, 36 (35.6%) indicated that their pregnancy was unintended. Of these 36 unintended pregnancies, 14 (38.9%) were mistimed, 20 (55.6%) were unwanted, and 2 (5.5%) occurred because the woman did not have a preference.

The rate of unintended pregnancies was significantly higher (69.4%) among the AGYWLHIV who had never used a male condom, compared to 30.6% among their counterparts who had used a male condom (X² = 4.073, df = 1, p = 0.044). Similarly, unintended pregnancies were more prevalent among those with no or only primary education (63.9%), compared to those with secondary education (19.4%) and tertiary education (16.7%) (X² = 6.653, df = 2, p = 0.036). Additionally, the unintended pregnancy rate was higher among YWLHIV who had never used dual protection methods (66.7%) compared to those who had used them (33.3%) (X² = 5.342, df = 1, p = 0.021).

### Use of the dual protection methods

The use of DPMs which typically involves combining a barrier method (such as the male or female condoms) with another modern contraception method (such as oral contraceptive pills, emergency contraceptive pills, implants, injectables or intrauterine devices) was reported by only five (1.2%) of the AGYWLHIV in the study (see Table 2). When broken down by the concordance or discordance status, only two concordant couples, two discordant couples and one indeterminate couple in the study reported the use of the DPMs that combines a barrier method with another modern contraceptive method.

Of the five AGYWLHIV using the DPMs, three (0.7%) participants were using implant in combination with the male condom, one (0.2%) participant was using the male condom in combination with emergency contraceptive pills (ECPs) and only one (0.2%) participant was using the male condom in combination with COC or POC pills before and or after sexual intercourse.

In fact, 212 (50.1%) of the participants reported using some form of modern family planning method against unintended pregnancy but not in combination with a barrier method for the protection against the HIV transmission to the male partners.

None of the AGYWLHIV (0%) reported using the female condoms in combination with a modern contraceptive method as a DPM. Therefore, the FCDPM use prevalence was 0% in this population. Similarly, none of the AGYWLHIV (0%) reported combining the use of modern contraceptive method with the taking of ARV drugs for PrEP or PEP by the HIV negative male partners as DPMs.

### Use of the female condoms alone

Of the 423 AGYWLHIV in the study, nobody (0%) reported ever using the female condom alone or alongside another modern contraceptive method for dual protection against both HIV transmission and unintended pregnancy.

### Use of the male condoms alone

Of the 423 AGYWLHIV in the study, the minority (124, 29.3%) reported ever using the male condom alone for the dual protection against both HIV transmission and unintended pregnancy. Furthermore, 138 (32.6%) of the AGYWLHIV reported using the male condom at their most recent sexual intercourse.

### Associations between the women’s socio-demographic, sexual and reproductive health factors, and the use of dual protection methods

The factors found to be significantly associated with the use of the DPMs shown Table 3 included the women’s marital status, period of sexual debut, pill burden per ART clinic drug refill visit, knowledge of the safer conception methods, and the use of the male condoms. The rest of the factors in the conceptual framework were examined but found to have no statistically significant associations with the use of the DPMs.

### Reasons for the non-use of the dual protection methods

The reasons for the non-use of the DPM among the AGYWLHIV in northern Uganda fall under five key themes.

### Dislike or disapproval of modern contraceptive methods

Many AGYWLHIV reported avoiding DPMs due to personal or partner disapproval of modern contraceptive methods. Some women feared their male partners’ opposition, with one participant expressing, *“My husband does not want. He threatened to divorce me should I ever get any family planning method”* (Participant 77, aged 24, gravida 3, para 3, and 0 abortions). Others had personal reservations, as highlighted by one young woman who simply stated, *“I don’t like family planning methods”* (Participant 93, aged 17, gravida 0, para 0, and 0 abortion).

### Lack of knowledge and misconceptions about dual protection methods

Many participants demonstrated knowledge gaps regarding DPMs, with some expressing unfamiliarity with these options. One participant admitted, *“I don’t really know much about family planning”* (Participant 307, aged 16, gravida 1, para 0, and 1 abortion), while another added, *“No one told me about them”* (Participant 195, aged 19, gravida 0, para 0, and 0 abortion).

Misconceptions also contributed to non-use, with some women mistakenly believing that the aspirin plus safe days can serve as a DPM. One participant mistakenly said, *“I am using Aspirin and safe days”* (Participant 214, aged 21, gravida 0, para 0, and 0 abortion).

Some young women also misunderstood the need for contraceptive use during certain reproductive stages. *“I am breastfeeding, have not yet seen my periods since childbirth and so do not need to use modern contraceptives”* (Participant 145, aged 24, gravida 2, para 2, and 0 abortion), one woman explained, demonstrating a misunderstanding of lactational amenorrhea. Others cited myths and fears, such as *“I fear myths about family planning methods like it causes cancer”* (Participant 87, aged 24, gravida 0, para 0, and 0 abortion). A few linked their non-use to concerns about HIV disclosure, as one woman shared, *“He kept telling me he was safe but never showed or told me his HIV test results”* (Participant 76, aged 21, pregnant, gravida 2, para 1, and 0 abortions).

### Reliance on the male condoms

Some AGYWLHIV relied solely on the male condoms for dual protection, believing them sufficient for preventing both unintended pregnancies and HIV transmission. One participant reasoned, *“No need [to use female dual protection methods] because my male partner uses male condoms”* (Participant 172, aged 18, gravida 0, para 0, and 0 abortion). Another similarly stated, *“I only use the male condoms to prevent both the transmission of HIV and unintended pregnancy”* (Participant 128, aged 21, gravida 0, para 0, and 0 abortion).

### Desire to conceive

The desire for pregnancy was another significant factor contributing to non-use. Some participants, particularly newly married women, prioritized conception over contraception. *“We are newly married and want to conceive a baby”* (Participant 221, aged 20, gravida 0, para 0, and 0 abortion), one participant explained. Others had existing children but desired more, as reflected by one woman’s statement: *“I want to conceive the third born baby”* (Participant 70, aged 24, pregnant, gravida 3, para 2, and 0 abortion).

### Fear of contraceptive side effects or stigma

Many participants cited concerns about side effects and stigma associated with hormonal contraceptive methods as the reasons for avoiding DPMs. One woman feared community judgment, stating, *“I am afraid of what other people in the community will say about me going to start family planning”* (Participant 152, aged 19, gravida 0, para 0, and 0 abortion). Others had personal experiences with side effects. *“I once used injectaplan. I had heavy bleeding and stopped using it. I cannot risk again”* (Participant 337, aged 24, gravida 2, para 2, and 0 abortion), one participant shared. Another added, *“I was experiencing a lot of side effects of over bleeding with pills. I cannot risk using modern contraceptive methods again”* (Participant 225, aged 18, gravida 0, para 0, and 0 abortion).

Additionally, concerns about unintended effects on pregnancy contributed to non-use. *“I fear modern contraceptive methods have side effects of abortion because I desire to get pregnant, but I get abortions”* (Participant 127, aged 22, gravida 1, para 1, and 0 abortion), one participant explained. Another voiced apprehension about contraceptive-related complications, stating, *“I am not using it [dual protection methods] because I hear people say modern contraceptive methods have so many negative effects such as bleeding and complications of pregnancy”* (Participant 124, aged 22, gravida 1, para 0, and 0 abortion).

## Discussions

### Prevalence of use of the FCDPM and other DPMs

The study reveals an alarmingly 0% usage of the FCDPM and only 1.2% usage of other forms of DPMs among the northern Ugandan AGYWLHIV population. This was extremely low usage rate compared to the 6% and 21.4%−28.8% dual contraceptive method prevalence found among southwestern Ugandan AGYWLHIV on ART and Ethiopian AGYWLHIV on ART respectively [29,32,33,49]. Worse still, the 1.2% DPM prevalence found in northern Ugandan AGYWLHIV population is much lower than the 18% − 40%, 29.6%, and the 27.2% prevalence rates reported among AGYWLHIV aged 16–49 years in Canada, Thailand, and Nigeria respectively [49,51,56]. This low uptake among the northern Ugandan AGYWLHIV suggests a substantial gap in the adoption of the DPMs. It also indicates that the current efforts to promote these methods as part of the comprehensive HIV prevention, treatment and care strategy are insufficient, highlighting the need for targeted interventions to increase awareness and improve access to these methods.

### Factors associated with the use of the DPMs

The study found that married Ugandan AGYWLHIV were more likely to use the DPMs. This finding concurs with previous studies conducted in Ethiopia [30,45,47]. It can be argued that marriage may provide an environment in which discussions around reproductive health and the use of DPMs can occur. Within marriage, women may have more consistent access to information, healthcare, and support systems, making them better informed to use the DPMs effectively. This highlights the role of marital stability and a regular partner in facilitating discussions and decisions about DPMs.

Furthermore, the northern Ugandan AGYWLHIV with lower pill burden were found to be more likely to use the DPMs than their counterparts with a higher pill burden. This finding is unique and suggests that the overall burden of a person’s medication regimen play a critical role in their ability to adopt and maintain additional preventive measures, such as DPMs. A lower pill burden—meaning fewer medications to take regularly—can make it easier for the AGYWLHIV to manage their daily routines, potentially reducing the physical and psychological strain associated with medication adherence. As a result, these women may be more open to incorporating DPMs into their health regimen, as they are not overwhelmed by their primary HIV treatment. The study underscores the importance of addressing the overall treatment burden for AGYWLHIV to improve adherence not only to ART regimens but also to DPMs. Reducing the complexity of HIV treatment regimens could have broader benefits for reproductive health outcomes, helping to ensure that these young women can effectively adopt DPMs to protect themselves and their male partners from both HIV transmission and unintended pregnancies.

More sexually experienced northern Ugandan AGYWLHIV were found to be more likely to use the DPMs compared to their counterparts who were sexually novice. This finding concurs with a previous study conducted in Ethiopia [47]. It suggests that sexually experienced AGYWLHIV may have more opportunities to interact with healthcare providers and receive advice about contraceptive methods during routine HIV care or other reproductive health visits. This implies that the sexual and reproductive health needs of the sexually experienced AGYWLHIV may be more visible within healthcare settings, while the needs of the sexually naïve individuals might go under-addressed. To bridge this gap, healthcare providers could ensure that discussions about sexual health and dual protection are integrated into the routine HIV care for all AGYWLHIV, regardless of their sexual history.

More so, the study found that the northern Ugandan AGYWLHIV with the correct knowledge about the safer conception methods were more likely to use the DPMs compared to their counterparts who didn’t know about the safer conception methods for WLHIV. This finding is unique to this study as the role of knowledge about safer conception methods wasn’t explored in the previous studies. Knowledge of safer conception provides a foundation upon which AGYWLHIV can make informed decisions about their sexual and reproductive health. This highlights the importance of integrating education about safer conception options into HIV care services, particularly for WLHIV who may wish to conceive in the future. Doing so could encourage a more proactive approach to protecting themselves and their male partners while planning their reproductive lives.

Furthermore, the study revealed that the northern Ugandan AGYWLHIV who have ever used the male condoms were more likely to use the DPMs compared to their counterpart who have never used the male condoms. This finding concurs with a previous study conducted in Brazil [36]. The association is attributable to the fact that the use of condoms whether male or female condom is an integral component of the DPM. It therefore underscores the importance of building on existing behaviours related to male condom use to promote the adoption of other forms of DPMs among the AGYWLHIV. By integrating education about both the male and female DPMs into the HIV prevention, treatment, care, and other sexual and reproductive health programs, healthcare providers can better support young women in managing their health and well-being.

### Reasons for non-use of the DPMs

The research finding that the northern Ugandan AGYWLHIV’s desire to conceive and produce children was one of the reasons for the non-use of DPMs warrants discussion. The negative association between the women’s or their male partner’s fertility desires and the use of DPMs was highlighted in the previous studies conducted in south-western Uganda [31] and Ethiopia [30,47,57]. Given that many AGYWLHIV may desire to conceive, health programs should incorporate education about safer conception methods into all sexual and reproductive health services. These safer conception methods include timed intercourse during ovulation, use of pre-exposure prophylaxis (PrEP) for the HIV negative partners, and assisted reproductive technologies, which can help them achieve their reproductive goals while minimizing the risk of HIV transmission. By providing information on these safer conception strategies, health programs can empower AGYWLHIV to make safe and informed choices about their reproductive health.

The study found that northern Ugandan AGYWLHIV were not using the DPMs due to the lack of knowledge about contraceptive methods generally, DPMs and the HIV transmission risks from unprotected sexual intercourse with male partners of undisclosed HIV status, unprotected sexual intercourse during pregnancy, exclusive breastfeeding, or safe days. These findings concur with previous studies conducted in Ethiopia [30,32,47,57], Nigeria [49], and Canada [29]. The implications of these research findings point to the critical need for comprehensive and targeted education about contraceptive methods, dual protection, and HIV transmission risks for AGYWLHIV in Uganda. By addressing knowledge gaps and providing accessible information, healthcare providers and policymakers can empower young women to make safe and informed choices about their sexual and reproductive health.

Fear of the side effects or stigma associated with the use of the hormonal contraceptives often used as a component of the DPMs emerged as barriers to the use of the DPMs among the northern Ugandan AGYWLHIV. This is a unique finding that wasn’t examined in the previous studies on the use of DPMs. The finding highlights the critical need for multifaceted approaches to address the barriers of fear of side effects and stigma associated with hormonal contraceptive component of the DPMs. By providing comprehensive education, fostering supportive environments, and offering a variety of modern contraceptive options, healthcare providers and policymakers can empower young women to make safe and informed decisions about their preferred modern contraceptive methods. This, in turn, can lead to increased use of the DPMs, reducing the risk of unintended pregnancies and HIV transmission, and improving the overall health outcomes for AGYWLHIV.

### Study strengths and limitations

The quantitative survey component of this mixed-method study that allowed for inclusion into the study of the various subgroups of the AGYWLHIV was a strength of the study. The sample size and the use of the probability sampling method alludes to the representative and generalizability of the study findings.

Nevertheless, the study wasn’t without limitations. One limitation was the very small sample (ten) for the pretest which was less than the recommended 10% of the sample size. Future studies should abide by the guidelines which stipulates using 10% of the sample size for pretest. The study was also premised on the assumptions that the female or male condoms only can guarantee dual protection which is challengeable as the condoms may break and sometimes the men or even the women themselves may distort their use during sexual intercourse. We also acknowledge that women may not have absolute control over the female condoms as its use will require dialogue, joint decision and cooperation from both sides of the partnership. Therefore, future studies preferably using qualitative methods should explore the level of control women can have over the female condoms as a female-only protection method. There was also lack of the perspectives of the health workers on the modern contraceptive methods available and offered from the health facilities especially the ART clinic where the participants usually obtain their ARV drug refills. This limitation was minimised by asking the participants multiple questions on modern contraceptive methods some of which were meant to validate the accuracy of their data.

The factors associated with the use of DPMs reported in the study could be confounded because the low DPMs use prevalence could not allow for further analysis for independent predictors. To minimize this limitation, a mixed-methods research design employed allowed for qualitative exploration of the reasons for non-use of the DPMs among the participants reporting non-use.

## Conclusions

The FCDPM use prevalence among the Ugandan AGYWLHIV was at 0% and that for any other form of DPMs was also extremely very low just at 1.2% compared to 29.3% for the use of male condom alone. Among the DPM users, the male condom was used alongside oral pills, emergency contraceptive pills or implant. The factors influencing the use of the DPMs include past experience in using the male condoms, knowledge about contraceptive methods, pill burden, woman’s fertility desires, knowledge and perceptions about dual protection and safer conception methods.

The ART programs should address female condom availability and access, leverage health education, addressing misconceptions, reducing fears about contraceptive side effects or stigma and counselling about modern contraceptives methods and the male condoms to promote the use of the DPMs including the FCDPMs. Women should be educated and counselled about the long-term reversible injectable, implants or IUD contraceptive methods to address the pill burden which affect the use of the dual protection methods.

## Supporting information

S1 File Data collection tool. S1 File AGYWLHIV is Adolescent girls and young women living with HIV; ART is antiretroviral therapy; ARV is antiretroviral; PLHIV is people living with HIV; PrEP is pre-exposure prophylaxis and PEP is post-exposure prophylaxis.
(PDF)
